# Clinicopathologic features of renal cell carcinomas seen at the Aga Khan University Hospital in Kenya

**DOI:** 10.3389/fmed.2022.981305

**Published:** 2022-11-08

**Authors:** Anderson Mutuiri, Samuel Gakinya

**Affiliations:** Pathology Laboratory, Department of Pathology, Aga Khan University Hospital, Nairobi, Kenya

**Keywords:** renal cell carcinoma, oncopathology, Kenya, genitourinary pathology, clear cell renal cell carcinoma

## Abstract

**Introduction:**

Kidney cancer accounted for 1. 8% of global cancer deaths according to Globocan 2020 estimates, with most of these being renal cell carcinomas. Lower rates of renal cell carcinoma are reported for Africa and these are expected to change for a combination of reasons. The clinical and morphologic characteristics of renal cell carcinoma seen within Kenya have not been described before. This study aims to partially fill this gap.

**Materials and methods:**

This was a cross-sectional descriptive study examining electronic histopathology reports from the Aga Khan University Hospital Nairobi Laboratory for the period January 2016 to May 2022.

**Results:**

Sixty cases of renal cell carcinoma were identified. The mean age at diagnosis was 55.3 years. The most common histologic subtype diagnosed was clear cell renal cell carcinoma (41.7%), followed by papillary renal cell carcinoma and renal cell carcinoma not further specified (both 21.7%), and chromophobe renal cell carcinoma (11.7%). The most frequent specimen type was resection, followed by cores of renal masses. The mean tumor size was 8.5 cm. Sixty-seven percent of patients presented with Stage III and above.

**Discussion:**

Renal masses were the commonest clinical indication for biopsy among the records reviewed. The male to female ratio, as well as the mean age at presentation were comparable to what is described in literature for other regions of the world. The proportions of the commonest histologic subtypes matched what is described in other parts of the world. Challenges in the identification of histologic subtypes included having a limited panel of antibodies for diagnosis and the lack of genetic molecular tests for histotyping.

**Conclusion:**

The spectrum of histologic subtypes of renal cell carcinoma seen at a tertiary referral hospital in Nairobi, Kenya was similar to that described in other parts of Africa and the globe. The age at presentation with renal cell carcinoma was consistent with what has been described in literature. Challenges were identified in the accurate histotyping of renal cell carcinoma due to constrained resources. Majority of cases diagnosed presented at advanced stage.

## Introduction

Kidney cancer is the 14th most common cancer by incidence and accounted for 1.8% of global deaths from cancer according to Globocan 2020 estimates (1), with most of these cancers being renal cell carcinomas (RCC). Globocan 2020 estimates that kidney cancer represented 1.1% of new cancers and 1.1% of deaths from cancer in Kenya (2). Most early-stage RCC are discovered on routine imaging for other diseases or patient complaints. Less than 20% of patients present with pressure (flank pain, abdominal fullness or swelling), urinary tract symptoms (bleeding, repeated infections from obstruction) or paraneoplastic syndromes (3). Of note, up to 20% of patients may present initially with metastatic disease to various sites (4, 5).

Risk factors for RCC include advancing age, male sex, smoking, and obesity. The global age standardized incidence rate for renal cancer per 100,000 is 6.1 for males and 3.2 for females (1). Dietary risk factors such as vegetable or meat intake have shown conflicting relationship with incidence of RCC in various studies (3). Acquired cystic kidney disease in patients on dialysis is also an established risk factor (6). Less commonly, rare genetic syndromes are associated with RCC including von Hippel-Lindau syndrome, Hereditary papillary renal carcinoma, Birt-Hogg-Dube syndrome, Succinate dehydrogenase (SDH)–deficiency, Hereditary leiomyomatosis and renal cell cancer and Hereditary renal carcinoma (HRC).

The highest incidence of renal cell carcinoma has been observed in Belarus and North America with lower rates reported among populations in Africa (3, 7). These reportedly lower rates are likely a reflection of disparities in access and provision of allopathic healthcare. One of the consequences of this is under-reporting because of inadequate infrastructure that leads to underperformance of cancer registries (4). Thus, this low incidence of RCC in Africa may represent an underestimation. In multiracial populations, it has been observed than individuals with African ancestry have an increased risk for RCC and worse outcomes (7). The World Health Organization (WHO) estimates that 1 in 5 adults and 1 in 10 children and teenagers will be obese by December 2023 in 10 high-burden African countries (8). There is already an established link between being overweight and obese and the risk for developing renal cell carcinoma. It is therefore expected that this increase in the number of obese and overweight individuals will lead to an increase in the incidence of RCC. It is also possible that the incidence of RCC will increase in sub-Saharan Africa, in the coming years, with increasing use of radiologic imaging for various other conditions as this becomes more available.

There are several distinct histologic subtypes of RCC that have been described, with distinct clinical behavior including presentation, aggressiveness, and response to targeted therapy (9). In addition, the current WHO 2022 classification of urinary and male genital tumors includes entities that are defined by molecular genetic abnormalities (9). This makes it challenging to accurately diagnose these tumor entities because the necessary molecular tests are not widely available, particularly so in low- and medium-income countries (LMICs).

The most common subtypes of RCC described include clear cell (75–85% of all RCC), papillary (10–15%) and chromophobe (5–10%). Of these, clear cell renal cell carcinoma tends to have a worse prognosis than most of the other subtypes, and a more aggressive clinical course. The other subtypes with bad prognosis are equivalent to the prognosis of clear cell renal cell carcinoma. Some of the rare histologic subtypes such as clear cell papillary RCC, have indolent behavior and this is now categorized as a tumor rather than carcinoma (9).

Management of renal cell carcinoma is dependent on the histologic subtype and the stage at disease presentation, with only surgery being curative for early and localized disease. There are several approved targeted therapies that may also be used in the adjuvant setting subsequently. Treatment of advanced renal cell carcinoma is supportive, although many new targeted treatments are under investigation (10, 11). Access to these targeted therapies remains a challenge in LMICs due to the prohibitive cost.

The Aga Khan University Hospital Nairobi Main Laboratory is part of a private nonprofit institution, the Aga Khan University Hospital. The anatomic pathology laboratory processes 14–16,000 surgical specimens annually, covering all areas of surgical pathology practice. The laboratory receives and reports specimens from the parent hospital as well as several private, faith-based and government institutions.

There is an epidemiological need to define the clinical and morphologic characteristics of renal cell carcinoma seen within Kenya specifically, and the East Africa region in general, as these data do not exist to the best of our knowledge. This clinicopathologic review aims to partially fill this gap. It is hoped that this information will inform policy and guide the allocation of resources such as training in oncology care, and the development and refinement of treatment strategies.

We aimed to describe the clinical and pathologic characteristics of renal cell carcinoma as seen at Aga Khan University Hospital (AKUHN) from January 2016 to May 2022. This description includes the presentation and diagnostic work up where available and/or relevant.

## Materials and methods

This was a cross-sectional descriptive study examining electronic histopathology reports from the Aga Khan University Hospital Nairobi Main Laboratory for the study duration. All renal cell carcinomas diagnosed in that period were included in the study. Data collected was entered in Microsoft Excel 2021 sheets. Data analysis was performed using Excel and SPSS version 20. The most relevant study limitation was finding incomplete reports.

## Results

Records were available for 60 patients with a histopathologic diagnosis of renal cell carcinoma, six of which were of biopsies from metastatic sites.

The clinical presentation was recorded for 45 cases. The commonest presentation of patients with RCC was a renal mass in 29 patients (64%, 95% CI 49–78), followed by flank pain in 8 patients (18%, 95% CI 8–32) and hematuria in 3 patients (6%, 95% CI 2–18). Many patients had more than one presentation recorded e.g., renal mass and hematuria and others had a single unique presentation such as metastasis to a unique site.

RCC was diagnosed with slightly higher frequency in males than females with a male to female ratio of 1.22:1.

**Figure 1:**
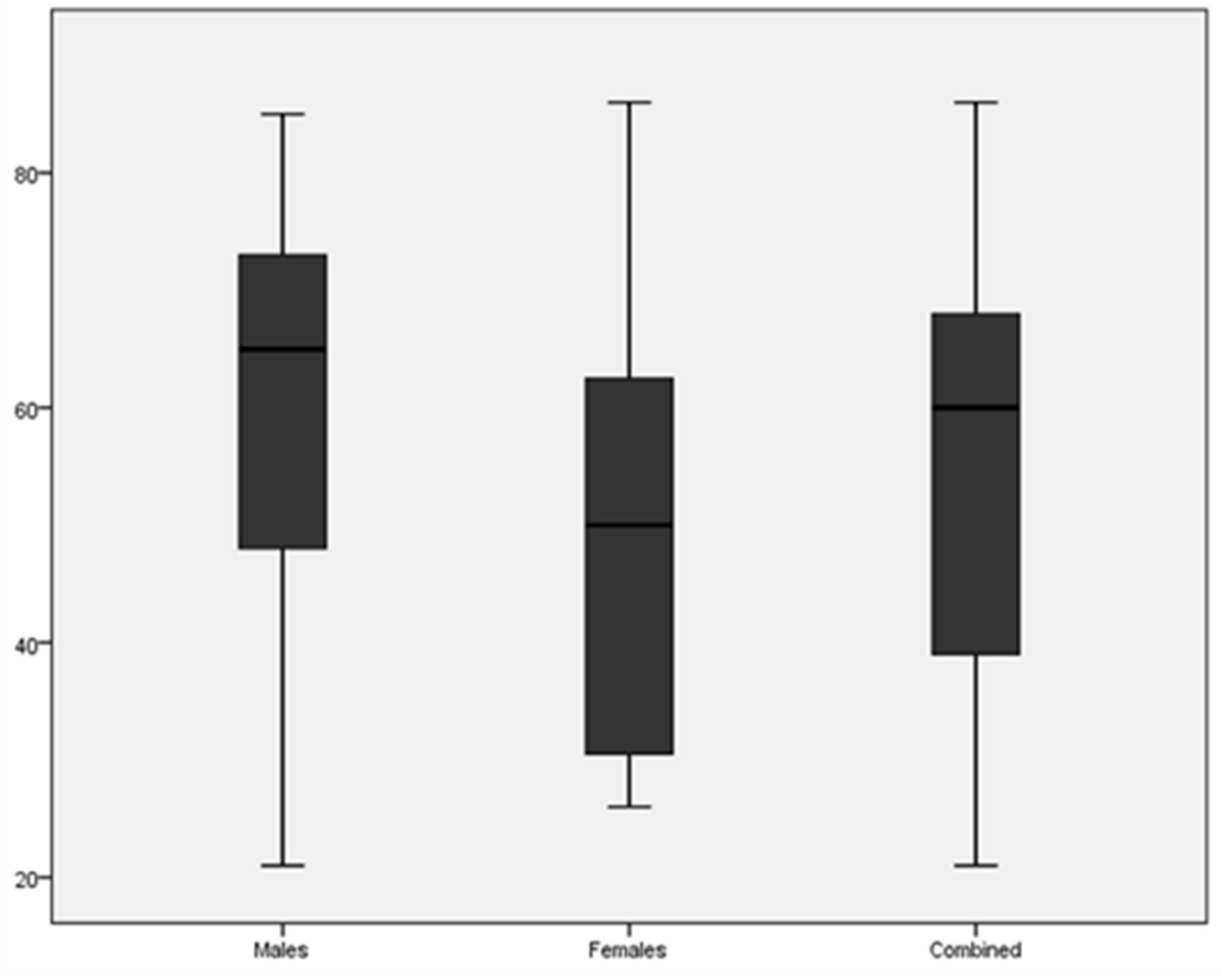
Boxplot comparing the age at diagnosis of renal cell carcinoma for males, females and both combined.

The average and median age at diagnosis of RCC are shown in Table 1 and compared in Figure 1. The mean age at presentation with renal cell carcinoma was older for males by 12 years compared to females.

**Table 1:** Age at diagnosis of renal cell carcinoma.

	**Number of patients**	**Youngest**	**Oldest**	**Mean**	**Std. Deviation**
Males	33	21	85	60.7	16.9
Females	27	26	86	48.7	18.4
Combined	60	21	86	55.3	18.5

**Figure 2:**
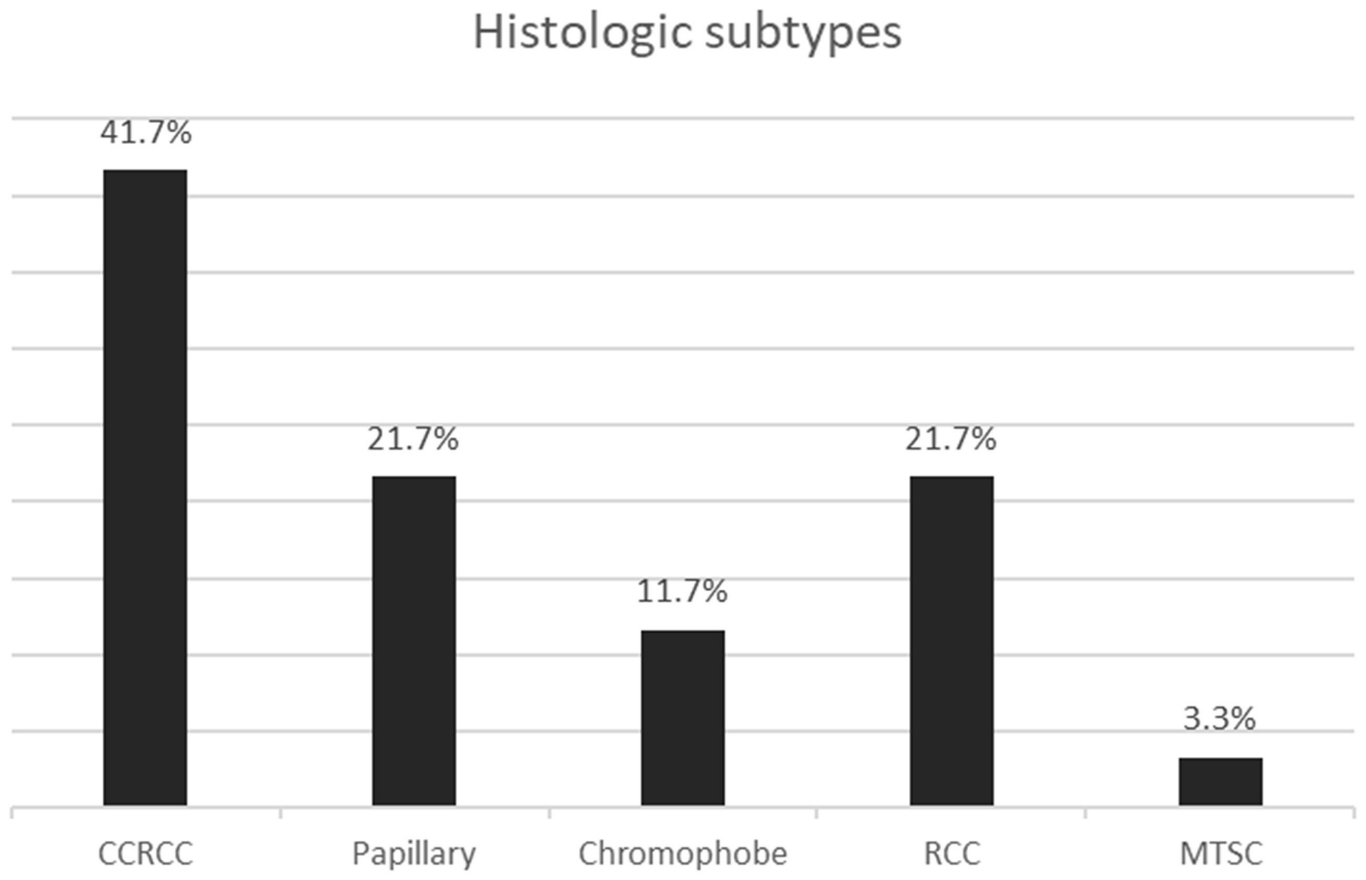
Bar graph showing the histologic subtypes of renal cell carcinoma. CCRCC, Clear cell; RCC, Renal cell carcinoma not otherwise specified; MTSC, Mucinous tubular and spindle cell.

The most common histologic subtype diagnosed was clear cell RCC. The next most frequent were papillary RCC and RCC not otherwise specified, as shown in Figure 2. Only two of the papillary RCCs were type 1, the rest being either type 2 or mixed type 1 and 2.

Tumor size was available for 32 resection specimens as shown in Table 2.

**Table 2:** Tumor size for resection specimens (*N* = 32).

	**Smallest**	**Largest**	**Mean**	**Median**	**95% CI**
Tumor size (cm)	3.5	18	9.7	8.5	1.4

The status of the margins was available for 29 of the resection specimens. Most (22 cases) had achieved clear margins. The positive margins were Gerota fascial margin (4 cases), renal vein (3 cases), renal sinus, perinephric fat, and parenchymal margins (1 of each).

Data on specimen type, tumor laterality, histologic grade, and stage of carcinomas are shown in Table 3. Only 3 cases had lymph nodes removed for staging.

**Table 3:** Data on specimen type, tumor laterality, histologic grade, and tumor stage.

**Variable**	**Value**	**Number (%, 95%CI)**
Specimen type (*N* = 60)	Nephrectomy (Simple or unspecified)	21 (35%, 23–48)
	Radical nephrectomy	13 (21.7%, 12–34)
	Renal core	16 (26.7%, 16–40)
	Partial nephrectomy	2 (3.3%, 0.4–12)
	Other core	5 (8.3%, 2.8–18)
	Other	3 (5%, 1–14)
Laterality (*N* = 49)	Right	25
	Left	24
Histologic grade (*N* = 39)	1	10 (26%, 13–42)
	2	18 (46%, 30–63)
	3	5 (13%, 4–27)
	4	6 (15%, 5–30)
Stage (AJCC prognostic groups) (*N* = 46)	I	9 (20%, 9–34)
	II	6 (13%, 5–26)
	III	14 (30%, 18–46)
	IV (Locally advanced)	6 (13%, 5–26)
	IV (Metastatic)	11 (24%, 13–39)

Twenty-seven of the cases had immunohistochemistry done either for histotyping or to identify the primary in cases of metastatic disease, based on clinical and imaging suspicion. The characteristics of these tumors are shown in Table 4.

**Table 4:** Clinicopathologic characteristics and immunoprofile of renal cell carcinomas.

**Case**	**Diagnosis**	**Age**	**Sex**	**Specimen**	**Pathologic Stage**	**Positive IHC stains**	**Negative IHC stains**
1	RCC	39	F	Scapular mass	M	AE1/AE3, Vimentin, EMA, E-cadherin	TTF1, HER2
2	Chromophobe	31	F	Nephrectomy	T2bNX	CK7	CD10, Vimentin, CD117, RCC, Inhibin
3	CCRCC	63	M	Mesenteric nodule	M	AE1/AE3, Vimentin, CD10	EMA, Desmin, S100, CD117
4	CCRCC	79	M	Renal core	CD10, Vimentin	
5	Chromophobe	27	F	Radical nephrectomy	T1bNX	CK7, RCC	Vimentin, CD10
6	CCRCC	63	F	Renal core	CD10, Vimentin	
7	RCC	60	M	Lung core	M	CD10	TTF1, CK7
8	RCC	60	F	Renal core	Vimentin	CK7
9	RCC	74	M	Renal core	CK7	Synaptophysin, RCC, CD10, Inhibin
10	Chromophobe	30	M	Radical nephrectomy	T1	CK7	Vimentin
11	Chromophobe	77	M	Renal core	CK7, CD117	Vimentin, CD10
12	Papillary	37	M	Renal core	CK7, AMACR, CD10	
13	CCRCC	30	M	Nephrectomy	T1bNX	Vimentin	CK7, CD10, RCC
14	Papillary	45	M	Nephrectomy	T1bNX	CD10, AMACR, CK7	
15	RCC	66	M	Renal core	CK7, EMA, Vimentin, CD10	CK20
16	MTSC	64	F	Nephrectomy	T2bNX	AE1/AE3, AMACR, CK7	CD10
17	Papillary	27	F	Renal core	M	AE1/AE3, AMACR, Vimentin	CK7, CD10
18	RCC	54	F	Liver core	M	CK7	CK20, Synaptophysin, HepPar1
19	RCC	85	M	Liver core	M	CK7, CD10,	HepPar1, p63
20	CCRCC	26	F	Nephrectomy	T3aNX	AE1/AE3, CK7, CD10, Vimentin	WT1, S100
21	RCC	42	M	Renal core	M	CD10, Vimentin, AMACR	CK7, CD117,
22	Chromophobe	65	F	Renal core	CK7, CD10	Vimentin
23	CCRCC	54	F	Nephrectomy	T1a	AMACR, Vimentin, CD10, weak AE1/AE3	CK7
24	Papillary	79	M	Renal core	AE1/AE3, CK7, Vimentin, AMACR	S100
25	Chromophobe	84	M	Radical nephrectomy	T3aN0	CK7, CD117	Vimentin
26	MTSC	48	M	Radical nephrectomy	T3aNX	CK7, AMACR	
27	Papillary	61	M	Renal core	CK7, Vimentin	S100, CD117

CCRCC, Clear cell renal cell carcinoma; RCC, Renal cell carcinoma, not otherwise specified; MTSC, Mucinous tubular and spindle cell RCC.

Three of the metastatic sites biopsied were liver, making this the commonest site of metastasis that had been biopsied. Other metastatic sites included a scapular mass, lung, and a mesenteric nodule.

## Discussion

A renal mass was the most common clinical presentation recorded in the reports, it is possible that the renal mass was found following investigation for the symptoms of abdominal fullness, pain, or hematuria. This sequence of preoperative clinical investigation was not necessarily captured in the request form accompanying the specimens submitted for evaluation.

The ratio of males to females in our study population was 1.22:1, which is compares to the described global ratio of 1.58:1, and therefore consistent with what is known that renal cell carcinoma has a slight male preponderance (1).

The mean age at which renal cell carcinoma was diagnosed was 55.3 years which is similar to what has been described in literature. More than 77% of kidney cancer patients are aged older than 50 years (12). The age also varied between men and women with males presenting at a slightly older age group, again similar to what has been described for global data (12).

The commonest histologic subtypes seen were similar in proportion to what has been described in other parts of the world and Africa in particular. It is noted that the current (2022) WHO Classification does not recommend grouping of papillary RCC into types 1 or 2, and the data presented represent the prior historical classification. Table 5 shows data from other studies in various parts of the African continent.

**Table 5:** Renal cell carcinoma clinicopathologic features in studies from Africa.

**References**	**Study type**	**Year**	**Location**	**Number of cases**	**Age (years)**	**Commonest RCC (%)**	**Pathologic Stage ≥3**
Amenu et al. (13)	Retrospective	2020	Ethiopia	64	Median 53.4	CCRCC 55, ChRCC 20, PRCC 18	34%
Atanda et al. (14)	Systematic review	2017	Nigeria	443	Mean 45 ± 4	CCRCC 60–85.7, PRCC 23.8–46.2	80–96%
Du Plessis et. al (15)	Prospective observational	2020	South Africa	31	Mean 56.39 (SD 10.16)	CCRCC 74, PRCC T2 16, PRCC Mixed 3, ChRCC 7	
Salako et al. (16)	Retrospective	2017	SW Nigeria	51	Median 41.7	CCRCC 60.8, ChRCC 17.6 PRCC13.7, Sarcomatoid 3.9, Mixed 1.9	52.9%

CCRCC, Clear cell RCC; PRCC, Papillary RCC; ChRCC, Chromophobe RCC.

There were challenges in the diagnosis of RCC that led to a proportion of tumors being categorized as RCC, not otherwise specified. Although the available panel of antibodies for immunohistochemistry was usually adequate to make a diagnosis of renal cell carcinoma, it was sometimes limited for histologic subtyping. The following antibodies were not available: carbonic anhydrase IX, TFE3, SDHB and TFEB. It should be noted that the latter three are used to diagnose very rare histologic subtypes. On the balance however, this may not always influence patient care adversely given that surgical resection is the mainstay of management for localized disease regardless of the histologic subtype.

Additionally, the current WHO Classification has a category of molecularly-defined RCC which cannot be diagnosed as such without the use of ancillary molecular genetic testing. Although in many instances there are morphologic clues to suggest some of these entities (papillary morphology in a young person and weak staining for keratin suggesting MiT family translocation RCC, flocculent cytoplasm suggesting SDH-deficient RCC), ultimately the definitive diagnosis requires molecular genetic testing and the availability of this remains a challenge. This becomes an area of potential future collaboration and capacity building, both in the performance and interpretation of molecular genetic testing. This capacity would be useful in molecular oncopathology of tumors from other sites as well, for diagnosis, prognosis and even therapy.

The other challenge seen with histologic typing is the changes in tumor morphology that would occur due to poor fixation of a large tumor mass. Several of the nephrectomy specimens were obtained from referring faith-based and government health facilities which were not nearby. This meant that some of the specimens had to be transported for several days before they were processed. If the entire nephrectomy specimen is placed in formalin without prior dissection, fixation may not be optimal and partial autolysis in an already large vascular-compromised mass led to challenges in morphologic assessment as well as immunohistochemical staining.

The surgical treatment was adequate in many cases, with most of the resections having clear margins. The positive margins were noted, as expected, in patients with advanced stage disease, and probably represent the challenge of adequate resection with large tumor burden. Surgical resection is curative treatment for early and localized disease in many instances and this probably contributes to the overall low mortality of renal cell carcinoma in that clinical context.

Thirty-four (57%) of the specimens on which a diagnosis of renal cell carcinoma was made were complete resections of the involved kidney, with only two of the specimens in our series being partial nephrectomies. If as expected there is a future increase in the diagnosis of smaller, earlier stage tumors, then we may expect to see more partial nephrectomy specimens. The other reason why there might be a possible change to more partial nephrectomies is the fact that several of the risk factors for RCC (chronic kidney disease, hypertension and obesity among others) also compromise kidney function (17). This means that there is greater need to perform nephron-sparing surgery as much as possible which is also the approach recommended in some current management guidelines (10).

The next most common specimen received was core biopsies of renal masses, both from patients with disease limited to the kidney as well as some patients with metastatic disease in order to confirm the primary. This specimen type would also be expected to increase in number, given the increasing role of renal mass biopsy in the diagnosis of renal cell carcinoma. Renal mass biopsy is increasingly being used to make decisions regarding whether to pursue active surveillance or proceed with surgery for localized renal masses suspicious for carcinoma (18).

Sixty-seven percent of the cases of RCC in our series were diagnosed at advanced stage (Stage III and above). Although this was similar to other data reported on the African continent in Table 5, this proportion was much higher than that reported in Euroamerica, for comparison, where only 25% of patients present with advanced disease (19).

## Conclusion

The spectrum of histologic subtypes of renal cell carcinoma seen at a tertial referral hospital in Nairobi Kenya matched that described in other parts of Africa and the globe. The age at presentation with renal cell carcinoma was comparable to what has been described in literature. A challenge was identified in the accurate histotyping of renal cell carcinoma caused by lack of access to molecular genetic testing. It is expected that there will be an increase in the number of partial nephrectomies and core biopsies received for diagnosis in order to plan patient management.

## Data availability statement

The raw data supporting the conclusions of this article will be made available by the authors, without undue reservation.

## Author contributions

All authors listed have made a substantial, direct, and intellectual contribution to the work and approved it for publication.

## Conflict of interest

The authors declare that the research was conducted in the absence of any commercial or financial relationships that could be construed as a potential conflict of interest.

## Publisher's note

All claims expressed in this article are solely those of the authors and do not necessarily represent those of their affiliated organizations, or those of the publisher, the editors and the reviewers. Any product that may be evaluated in this article, or claim that may be made by its manufacturer, is not guaranteed or endorsed by the publisher.

## References

[1] Sung H Ferlay J Siegel RL Laversanne M Soerjomataram I Jemal A. Global cancer statistics 2020: GLOBOCAN estimates of incidence and mortality worldwide for 36 cancers in 185 countries. CA Cancer J Clin. (2021) 71:209–249. 10.3322/caac.21660 33538338

[2] World Health Organization International Agency for Research in Cancer. Global Cancer Observatory. Available online at: https://gco.iarc.fr/today/data/factsheets/populations/404-kenya-fact-sheets.pdf.

[3] Padala SA Barsouk A Thandra KC Saginala K Mohammed A Vakiti A. Epidemiology of renal cell carcinoma. World J Oncol. (2020) 11:79–87. 10.14740/wjon1279 32494314 PMC7239575

[4] Klaassen Z Sayyid RK Wallis CJD. Lessons learned from the global epidemiology of kidney cancer: a refresher in epidemiology 101. Eur Urol. (2019) 75:85–87. 10.1016/j.eururo.2018.09.035 30274700

[5] National Cancer Institute. Cancer Facts. Available online at: https://seer.cancer.gov/statfacts/html/kidrp.html.

[6] Brennan JF Stilmant MM Babayan RK Siroky MB. Acquired renal cystic disease: implications for the urologist. Br J Urol. (1991) 67:342–8. 10.1111/j.1464-410X.1991.tb15158.x 2032071

[7] Chow WH Dong LM Devesa SS. Epidemiology and risk factors for kidney cancer. Nat Rev Urol. (2010) 7:245–57. 10.1038/nrurol.2010.46 20448658 PMC3012455

[8] WHO Africa,. Obesity Rising in Africa, WHO Analysis Finds. (2022). Available online at: https://www.afro.who.int/news/obesity-rising-africa-who-analysis-finds.

[9] Raspollini MR Moch H Tan PH. Renal cell tumors: introduction. In:WHO Classification of Tumours, Editorial Board. Urinary and male genital tumours [Internet]. 5th ed. Lyon: International Agency for Research on Cancer (2022). Available online at: https://tumourclassification.iarc.who.int/chapters/36.

[10] [Guideline] National Comprehensive Cancer Network. NCCN Clinical Practice Guidelines in Oncology. Kidney Cancer. NCCN (2022). Available online at: https://www.nccn.org/professionals/physician_gls/pdf/kidney.pdf (accessed June 23, 2022).

[11] Pontes O Oliveira-Pinto S Baltazar F Costa M. Renal cell carcinoma therapy: current and new drug candidates. Drug Discov Today. (2022) 27:304–14. 10.1016/j.drudis.2021.07.009 34265458

[12] Bai X Yi M Dong B Zheng X Wu K. The global, regional, and national burden of kidney cancer and attributable risk factor analysis from 1990 to 2017. Exp Hematol Oncol. (2020) 9:27. 10.1186/s40164-020-00181-3 33005476 PMC7525971

[13] Amenu R Seyoum N Deneke A. Clinical presentation and pathologic patterns of renal cell carcinoma at a tertiary hospital in Addis Ababa, Ethiopia: a retrospective study. East Central Afr J Surgery. (2020). Available online at: https://journal.cosecsa.org/index.php/ECAJS/article/view/20180053/1547#info

[14] Atanda AT Haruna MS. Renal cell carcinoma in Nigeria: a systematic review. Sahel Med J. (2017) 20:137–42. 10.4103/smj.smj_67_16

[15] Du Plessis DE Van Deventer H Fernandez P Van Der Merwe A. A prospective observational study of the epidemiology and pathological profile of RCC in a South African referral centre. Afr J Urol. (2020) 26:15. 10.1186/s12301-020-00022-z

[16] Salako AA Badmus TA Badmos RA David RA Laoye A Akinbola IA. Renal cell carcinoma in a semi-urban population of South-Western Nigeria. East Afr Med J. (2017) 94:1. Available online at: https://www.ajol.info/index.php/eamj/article/view/155005

[17] Lipworth L Tarone RE McLaughlin JK. Renal cell cancer among African Americans: an epidemiologic review. BMC Cancer. (2011) 11:133. 10.1186/1471-2407-11-133 21486465 PMC3087713

[18] Williamson SR. The expanding role of renal mass biopsy. Diagn Histopathol. (2019) 25:379–89. 10.1016/j.mpdhp.2019.07.003

[19] Atkins MB. Clinical manifestations, evaluation, and staging of renal cell carcinoma. In: Richie JP, editor. UpToDate. Available online at: https://www.uptodate.com/contents/clinical-manifestations-evaluation-and-staging-of-renal-cell-carcinoma#H23 (accessed June 23, 2022).

